# Mapping intellectual structure and research hotspots in the field of coronary heart disease and apoptosis: A bibliometric analysis from 1994 to 2025

**DOI:** 10.1097/MD.0000000000045766

**Published:** 2025-11-07

**Authors:** Wanjun Jin, Qi Yu, Siqiao Wang

**Affiliations:** a Tongji Hospital Affiliated to Tongji University, Tongji University, School of Medicine, Shanghai, China; b University of Melbourne, Parkville, Australia; c Chengdong College, Northeast Agricultural University, Harbin, China.

**Keywords:** apoptosis, bibliometrics, cell death, coronary heart disease, research trends

## Abstract

**Background:**

Apoptosis plays a significant role in the pathogenesis of coronary heart disease (CHD). This study investigated the research status and hotspots in the field of apoptosis and CHD.

**Methods:**

This bibliometric analysis analyzed all articles and reviews from Web of Science Core Collection (dated July 7, 2025), using quantitative and visual mapping approaches. We employed the Bibliometrix R package to conduct a thorough analysis of the selected literature, which provided the hot topics, academic collaborations, and trends within the field.

**Results:**

We identified 5650 publications related to apoptosis and CHD, and the number of related articles has increased significantly in recent years. These publications came from 23,645 authors, and Zhang Y was the most influential author in this field. The USA had the highest impact and international collaboration. Apoptosis is activated via distinct pathways and exhibit interactions via Beclin 1-Bcl-2/Bcl-xL complex, mTOR, TRAIL, TNF-α, endoplasmic reticulum (ER) stress, and the p53 pathway. Excessive apoptosis may exacerbate myocardial ischemia, ischemia/reperfusion (I/R) injury, postischemic cardiac remodeling, and coronary atherosclerosis, excluding the progression of atherosclerosis induced by vascular smooth muscle cells. Given the pathological significance of apoptosis in CHD, there is a clear therapeutic potential in targeting apoptosis. Certain pharmacological agents, including mTOR inhibitors and AMPK activators, have been identified as regulators of apoptosis. The apoptosis-related cellular and molecular mechanisms in the progression and treatment of CHD have been the hot topics in this field.

**Conclusion:**

This study provided researchers with an overview of emerging trends in the field of apoptosis and CHD. Future studies should elucidate the roles of novel biomarkers, develop multi-target therapeutic strategies, and explore the interactions between apoptosis and other cellular processes.

## 1. Introduction

Coronary heart disease (CHD) represents the primary contributor to mortality associated with cardiovascular conditions.^[1]^ The fundamental pathology of CHD is characterized by the impairment of myocardial and vascular functions, which are instigated and aggravated by factors such as oxidative stress, inflammatory responses, and apoptosis.^[2]^ Recent studies have increasingly highlighted the role of apoptosis in the pathogenesis of CHD,^[3,4]^ with a growing number of scholars focusing on the apoptosis-related molecular and cellular mechanisms.^[5,6]^ Notably, apoptosis is classified as type 1 programmed cell death, featured by the initial disintegration of cytoskeleton, while the integrity of organelles is maintained until the later phases of the process.^[7]^ Apoptosis serves as adaptive responses that are initially crucial for cellular survival, growth, and homeostasis. Importantly, apoptosis functions as a significant regulator in CHD,^[8]^ a topic that has garnered increasing attention in contemporary research.

The specific mechanisms by which apoptosis influences CHD are being elucidated. However, the overarching thematic areas remain ambiguous. Current evidence regarding the role of apoptosis in CHD is not yet fully comprehended. Although numerous studies have been conducted on apoptosis and CHD, there has been an absence of bibliometric analyses in this field. The relevant research has been disseminated across various journals, complicating the identification of the most impactful literature and trending topics within this field. A thorough bibliometric analysis regarding this research field has yet to be conducted, which would enable researchers to quickly grasp the landscape and pinpoint significant research hotspots. Consequently, the present study aimed to address this gap through employing bibliometric methods to explore the emerging trends and key topics within this area of research.

## 2. Materials and methods

### 2.1. Data acquisition and retrieval strategies

The Web of Science Core Collection (WOSCC) database was chosen as the data source in this study.^[9–11]^ The retrieval strategies were (TS = “coronary artery disease” OR TS = “coronary heart disease” OR TS = “acute coronary syndrome” OR TS = “ischemic heart disease” OR TS = “acute myocardial infarction” OR TS = “ST-elevated myocardial infarction” OR TS = “non-ST-elevated myocardial infarction”) AND (TS = “apoptosis”).^[12,13]^ All articles and reviews published in English were collected between 1994 and 2025.

### 2.2. Data analysis

The bibliometric method has been extensively employed in the analysis of literature to assess critical information derived from published works.^[14]^ By examining various elements such as authors, journals, institutions, countries, and keywords, we provided a comprehensive understanding of the evolutionary trends as well as focal points within a specific field.^[15]^ Bibliometrix is a powerful software tool for conducting bibliometric analyses.^[16]^ The bibliometric analysis was conducted by utilizing Bibliometrix in R.

## 3. Results

### 3.1. Analysis of annual publications

The initial literature search resulted in the identification of 5892 documents published between January 1st, 1994, and July 7th, 2025 sourced from the WOSCC. Following the application of established inclusion and exclusion criteria, a total of 5650 articles and reviews were downloaded for further analysis. Consequently, this study encompasses 5650 documents published from 1994 to 2025, authored by 23,645 individuals across 1259 journals. The average citation count per document was 47.57, with an aggregate of 216,673 references cited throughout all articles and reviews.

**Figure 1.**
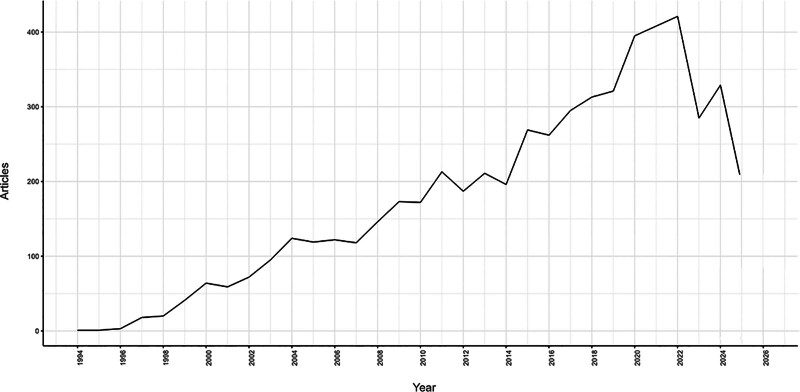
Annual publications in apoptosis and coronary heart disease from 1988 to 2024. A polynomial function *y* = 0.0875 × 2 − 338.1*x* + 326187 (*R*^2^ = 0.9385, *x* is the year, *y* is the annual number of publications) was used to model the annual publication trend.

The annual publications in the field of apoptosis and CHD, as revealed by Figure 1, were sporadic and fewer than 100 before the year 2003. The publication number began to show quick growth after 2007 and peaked at over 400 outputs in 2022. However, the number of publications has decreased in 2023. The data retrieval date (7th July 2025) was the decline of publications in this study. Further, a polynomial function *y* = 0.0875 × 2 − 338.1*x* + 326187 (*R*^2^ = 0.9385, *y* is the annual publication number, and *x* is the year) was developed to model the annual publication trends. The publication number were predicted to reach around 339 in 2025.

### 3.2. Analysis of countries, affiliations, and authors

**Figure 2.**
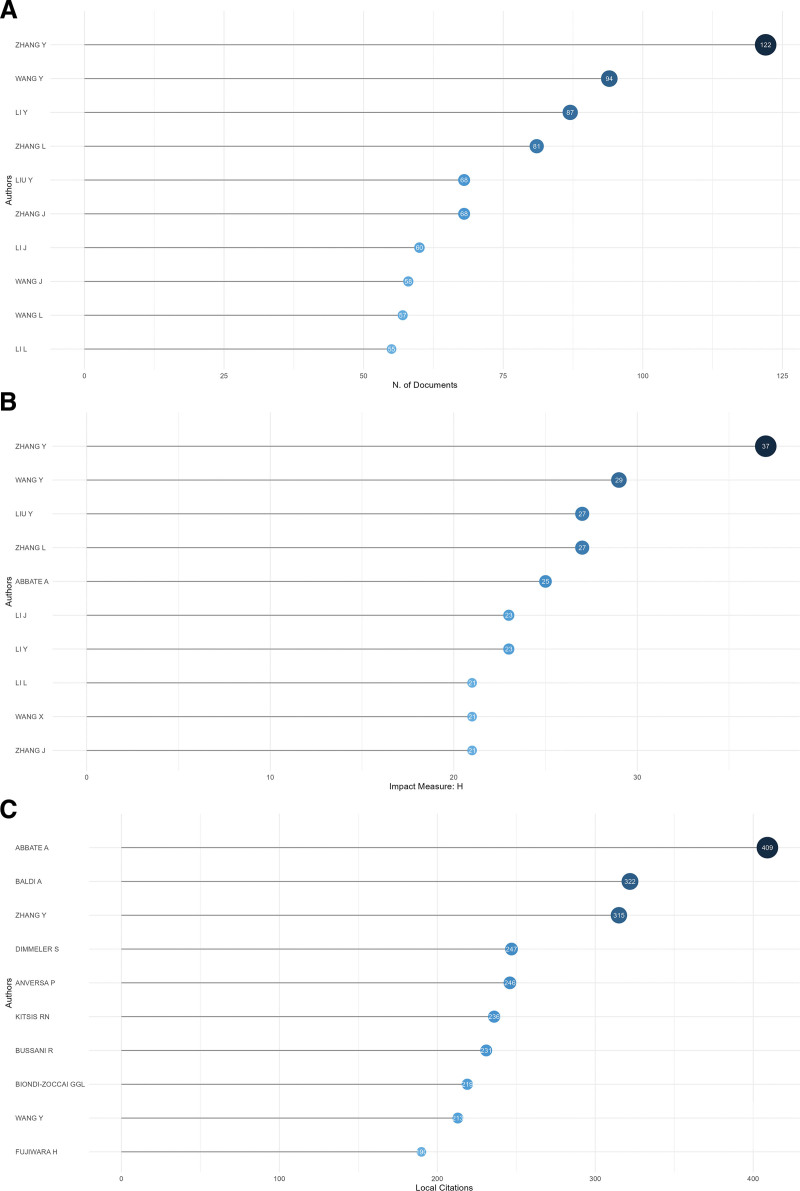
Analysis of authors. (A) The top 10 most relevant authors who have published most of the articles in the apoptosis and coronary heart disease were listed accordingly. (B) The top 10 authors’ local impact measured by H-index, which means that an author has published H articles and each of them has been cited for at least H times. (C) The top 10 most local cited authors, and local citation of a document meant that it was cited by any other document in our retrieval collection.

As of the retrieval date, July 7th, 2025, a total of 23,645 authors have contributed articles within this domain. Analyzing the most prolific and influential authors can provide valuable insights for researchers seeking to identify significant areas for further investigation. The top 10 authors, each having published a minimum of 50 articles, are presented in Figure 2A. Notably, “Zhang Y” and “Wang Y” lead the list with 122 and 94 publications, respectively, thereby surpassing their peers, as detailed in Table 1. The H-index metric indicates that an author has H articles, each of which has been cited more than H times,^[17]^ which can provide a concurrent assessment of both the quality and quantity of publications produced by scholars. Among the top 10 authors, the H-index ranged from a minimum of 21 to a maximum of 37 (Fig. 2B). Furthermore, documents that were cited by other works within the retrieval collection were classified as local citations. Authors and documents that exhibited a significant number of local citations were indicative of their considerable impact in the field. Notably, the author “Abbate A” showed the highest number of local citations, totaling 409, while the remaining 9 authors each garnered over 190 local citations (Fig. 2C). With a total of 122 articles and 315 citations, “Zhang Y” emerged as the leading author across all statistical metrics, achieving an H-index of 37, thereby establishing “Zhang Y” as the most influential figure in this field.

**Table 1:** The top 10 most relevant authors ranked by fractionalized frequency.

Rank	Author	Fractionalized frequency	Articles
1	Zhang Y	16.09	122
2	Wang Y	14.13	94
3	Li Y	12.64	87
4	Zhang L	10.72	81
5	Liu Y	9.80	68
6	Zhang J	8.69	68
7	Li J	7.96	60
8	Wang J	9.28	58
9	Wang L	7.44	57
10	Li L	7.66	55

**Figure 3.**
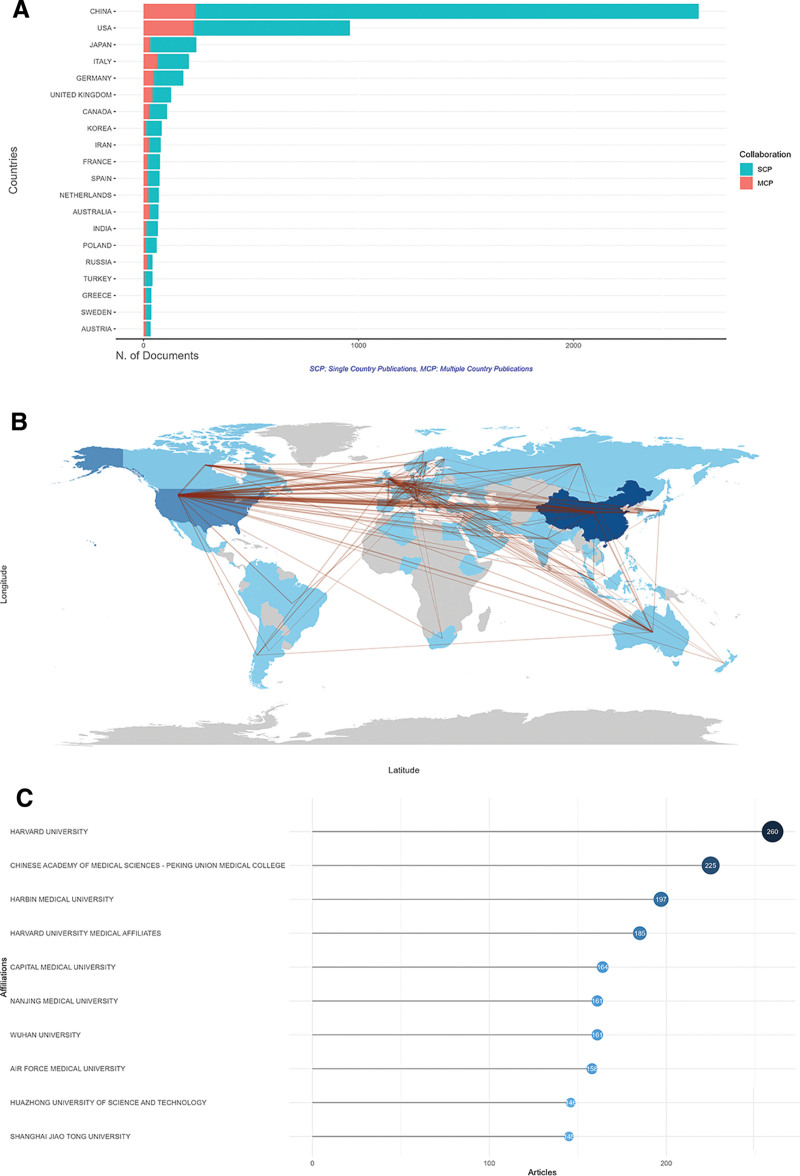
Analysis of countries and institutions. (A) The top 20 productive countries which were measured by the number of documents written by corresponding authors’ countries. SCP indicated that the investigation was finished by only one country, while MCP indicated that the researches were conducted by multiple countries. (B) Visualization of collaboration among countries by a network map; the line number means the cooperation times between countries, and the color density is proportional to the total publications. (C) The top 10 affiliations with the most publication outputs.

The top 3 countries in scientific production included China, the United States, and Japan. China published 2582 articles (45.70 %), followed by the United States with 960 articles (16.99 %), and Japan with 246 articles (4.35 %) (Table 2). Further, the countries/regions’ collaboration and distribution of scientific publications were shown in Figure 3A. China, the USA, and Italy ranked the top 3 countries with the highest multiple-countries publications (Table 3). Collaborative networks showed the cooperation degree among different countries (Fig. 3B). China showed the highest level of cooperation with the USA, Australia, and the United Kingdom. Furthermore, the top 10 most cited countries are displayed in Table 4. The top 3 most cited countries were the United States (n = 83,124), China (n = 63,469), and Germany (n = 17,304). The top 10 most productive institutions are illustrated by Figure 3C, and the Harvard University ranked the first (n = 260).

**Table 2:** The top 10 most productive countries.

Rank	Country	Publications	Proportion (%)
1	China	2582	45.7
2	USA	960	17.0
3	Japan	246	4.4
4	Italy	211	3.7
5	Germany	185	3.3
6	UK	128	2.3
7	Canada	109	1.9
8	Korea	84	1.5
9	Iran	80	1.4
10	Spain	76	1.3

**Table 3:** The top 10 most cooperative countries/regions.

Rank	Country	SCP	MCP	MCP (%)
1	China	2340	242	9.4
2	USA	727	233	24.3
3	Japan	215	31	12.6
4	Italy	147	64	30.3
5	Germany	136	49	26.5
6	UK	86	42	32.8
7	Canada	83	26	23.9
8	Korea	72	12	14.3
9	Iran	55	25	31.3
10	Spain	56	20	26.3

MCP = multiple-country publications, SCP = single-country publications.

**Table 4:** The top 10 most cited countries/regions.

Rank	Country	TCs	AACs
1	USA	83,124	86.60
2	China	63,469	24.60
3	Germany	17,304	93.50
4	Japan	14,006	56.90
5	UK	13,494	105.40
6	Italy	11,289	53.50
7	Canada	9135	83.80
8	France	8575	112.80
9	Netherlands	4722	66.50
10	Australia	4110	59.60

AACs = average article citations, TCs = total citations.

### 3.3. Analysis of journals

**Figure 4.**
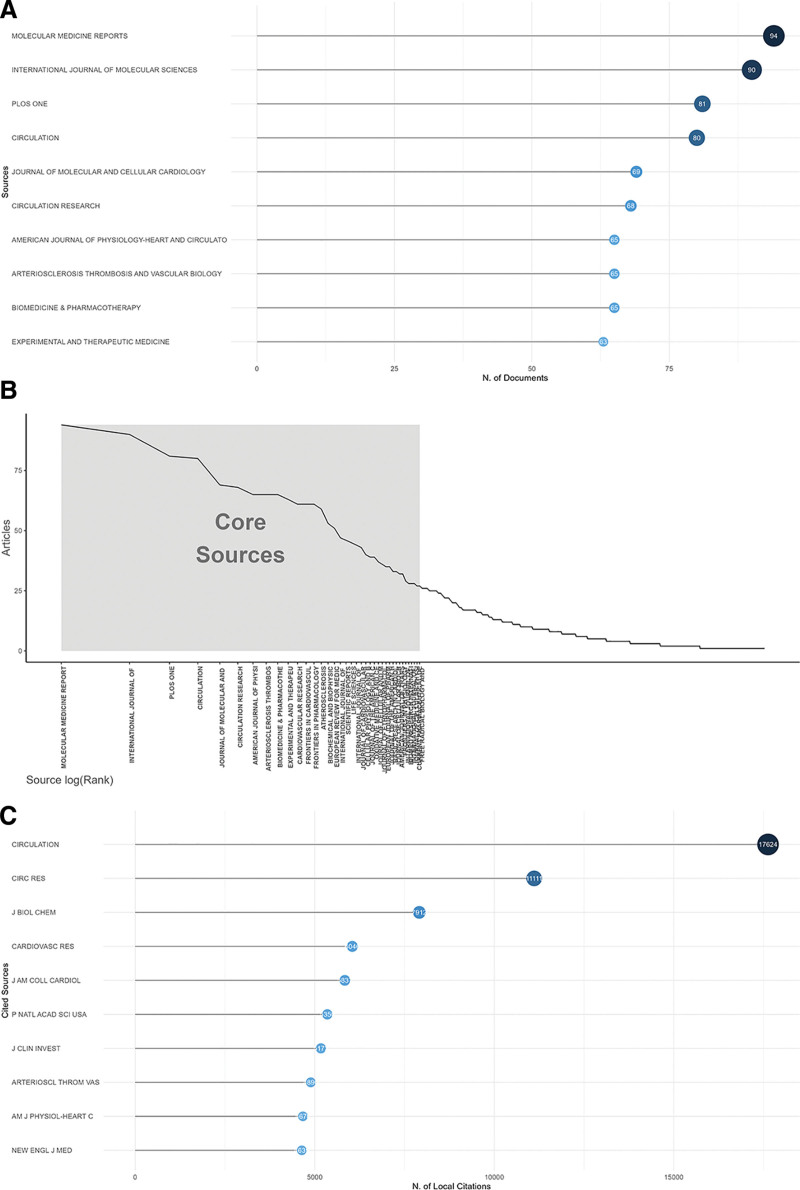
Analysis of journals. (A) The top 10 most relevant journals. (B) Journals’ order of arrangement corresponded to their publication outputs. All of the 19 most relevant journals were among the core sources, meaning that they were all pronounced in this area. (C) The top 20 most local cited journals.

A total of 1259 journals have published related articles in the research field. Figure 4A shows the top 10 most productive journals. According to the Branford law, these journals were located in the core zone (Fig. 4B). As measured by H-index, *Circulation* (H-index = 62), *Circulation Research (CIRC RES*, H-index = 58), and *Journal of Molecular and Cellular Cardiology* (H-index = 42) ranked the top 3 most influential journals. Further, the top 10 most locally cited journals are shown in Figure 4C. The top 3 most cited journals were *Circulation* (n = 17,624), *Circulation Research* (n = 11,111), and *Journal of Biological Chemistry* (n = 7912).

### 3.4. Analysis of cited documents

**Figure 5.**
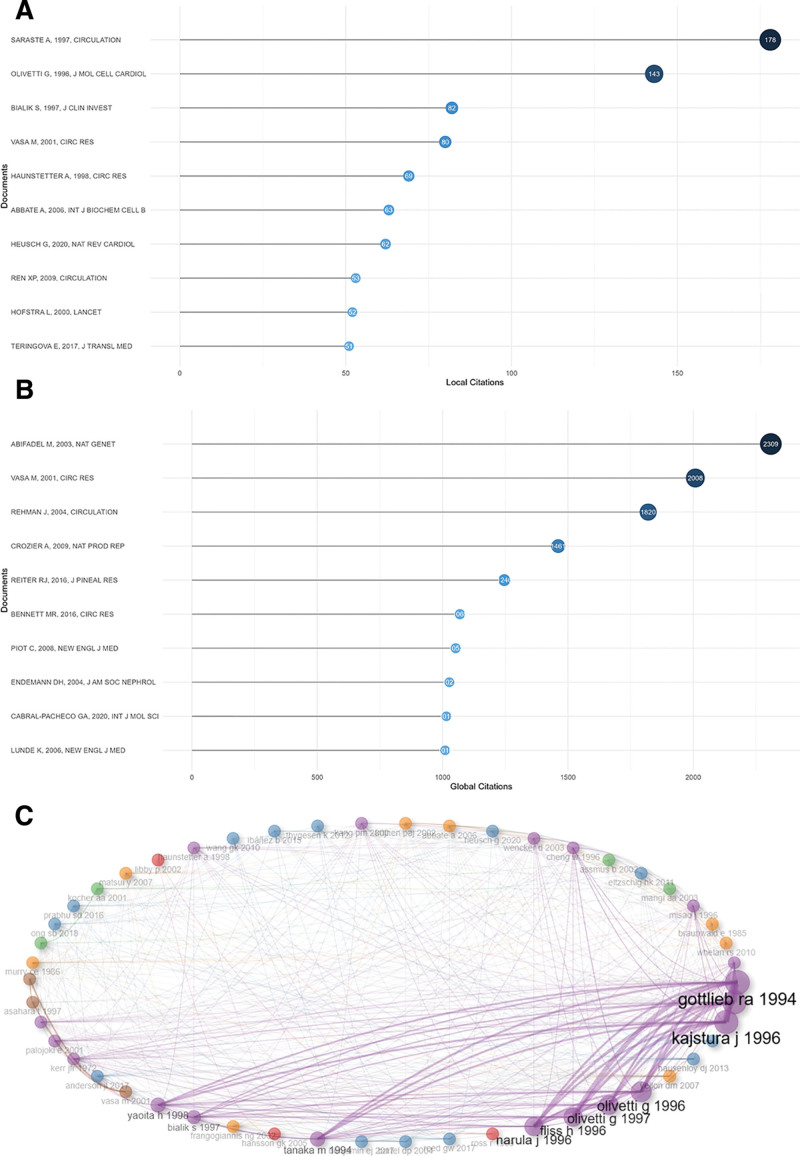
Analysis of documents. (A) The top 10 most local cited documents. Documents meant the publications that we have downloaded from Web of Science core database with our retrieval text. (B) The top 10 most global cited documents. (C) Documents clusters. Documents with high co-citation relationships were gathered together.

The top 10 locally and globally cited documents are shown in Figure 5A and B, respectively. Local citation suggested the documents were cited in the field of apoptosis and CHD (Table 5). Moreover, global citation of documents showed that it was cited by other documents in distinct academic fields.

**Table 5:** The top 10 most local cited documents.

Rank	Title	Author	Year	Journal	LCs
1	Apoptosis in human acute myocardial infarction	Saraste A	1997	Circulation	178
2	Acute myocardial infarction in humans is associated with activation of programmed myocyte cell death in the surviving portion of the heart	Olivetti G	1996	J Mol Cell Cardiol	143
3	Myocyte apoptosis during acute myocardial infarction in the mouse localizes to hypoxic regions but occurs independently of p53	Bialik S	1997	J Clin Invest	82
4	Number and migratory activity of circulating endothelial progenitor cells inversely correlate with risk factors for coronary artery disease	Vasa M	2001	Circ Res	80
5	Apoptosis: basic mechanisms and implications for cardiovascular disease	Haunstetter A	1998	Circ Res	69
6	Acute myocardial infarction and heart failure: role of apoptosis	Abbate A	2006	Int J Biochem Cell B	63
7	Myocardial ischemia–reperfusion injury and cardioprotection in perspective	Heusch G	2020	Nat Rev Cardiol	62
8	MicroRNA-320 is involved in the regulation of cardiac ischemia/reperfusion injury by targeting heat-shock protein 20	Ren XP	2009	Circulation	53
9	Visualisation of cell death in vivo in patients with acute myocardial infarction	Hofstra L	2000	Lancet	52
10	Apoptosis in ischemic heart disease	Teringova E	2017	J Transl Med	51

The most locally cited document, “*Apoptosis in Human Acute Myocardial Infarction*,” by Saraste et al^[18]^ (178 local citations), demonstrated that a subset of myocytes undergoes apoptosis and pronounced necrosis in response to ischemia-reperfusion injury. It indicated that the modulation of apoptosis may represent a unique approach for cardio-protection in acute myocardial infarction (AMI) of humans. The second-highest locally cited document, “*Acute myocardial infarction in humans is associated with activation of programmed myocyte cell death in the surviving portion of the heart*,” by Olivetti et al^[19]^ (143 local citations), highlighted that apoptosis was a critical complicating factor in AMI, as it can exacerbate the extent of myocyte cell death associated with coronary artery occlusion.^[19]^ The third most locally cited document, “*Myocyte* apoptosis during acute myocardial infarction in the mouse localizes to hypoxic regions but occurs independently of p53,” by Bialik et al^[20]^ (82 local citations), highlighted the necessity of p53 for myocyte apoptosis during myocardial infarction. Their findings indicated the presence of a p53-independent mechanism that facilitates myocyte apoptosis in myocardial infarction.

The highest globally cited document, “*Autosomal-dominant hypercholesterolemia due to PCSK9 gene mutation*” by Abifadel et al^[21]^ (2309 global citations), identified a third locus linked to alcohol dependence and harm, specifically HCHOLA3 located at 1p32. They presented 2 mutations in the PCSK9 gene, which encodes proprotein convertase subtilisin/kexin type 9, that are implicated in the etiology of alcohol dependence and harm. The PCSK9 gene is responsible for encoding NARC-1 (neural apoptosis regulated convertase), a recently discovered human subtilase that is predominantly expressed in the liver, which plays as a risk factor for CHD. The second-highest globally cited and the highest locally cited document, “*Effect of Cyclosporine on Reperfusion Injury in Acute Myocardial Infarction*,” by Piot et al^[22]^ (1052 global citations), identified that the administration of cyclosporine during reperfusion may be linked to a reduction in infarct size, as indicated by certain metrics, compared to placebo. These findings are preliminary and necessitate validation through a larger clinical trial. The third highest globally cited document, “Secretion of angiogenic and antiapoptotic factors by human adipose stromal cells,” by Rehman et al^[23]^ , elucidated the angiogenic and anti-apoptotic capabilities of readily obtainable subcutaneous adipose stromal cells by showcasing their secretion of various synergistic proangiogenic growth factors. It indicated the autologous administration of either native or genetically modified subcutaneous adipose stromal cells, which were influenced by hypoxic conditions, could serve as a novel therapeutic strategy for promoting angiogenesis or providing cardiovascular protection.

In Figure 5C, the articles and reviews were categorized into 4 distinct clusters based on the keywords plus, which includes both the authors’ keywords and those supplemented by the WOSCC. The purple and blue clusters emerged as the most significant. Within these clusters, the highly cited documents indicated that apoptosis represented a particular characteristic of reperfusion injury in cardiac myocytes, ultimately resulting in delayed cell death.^[24]^ Apoptosis may provide an important target for cardio-protection during evolving acute myocardial-infraction in humans.^[18]^ These studies laid the foundation in the research field. In recent years, the examination of the functions of novel apoptosis-related biomarkers of CHD, including the long noncoding RNAs (lncRNAs) and microRNAs (miRNAs), has yielded significant advancements in understanding the pathophysiological mechanisms associated with acute myocardial ischemia-reperfusion injury.^[25]^ These studies have also led to the identification of novel biomarkers and therapeutic strategies for the detection and management of CHD. Both pharmacological and genetic modifications of these biomarkers exhibited promising therapeutic potential to enhance clinical outcomes for patients with CHD.

### 3.5. Analysis of keywords

**Figure 6.**
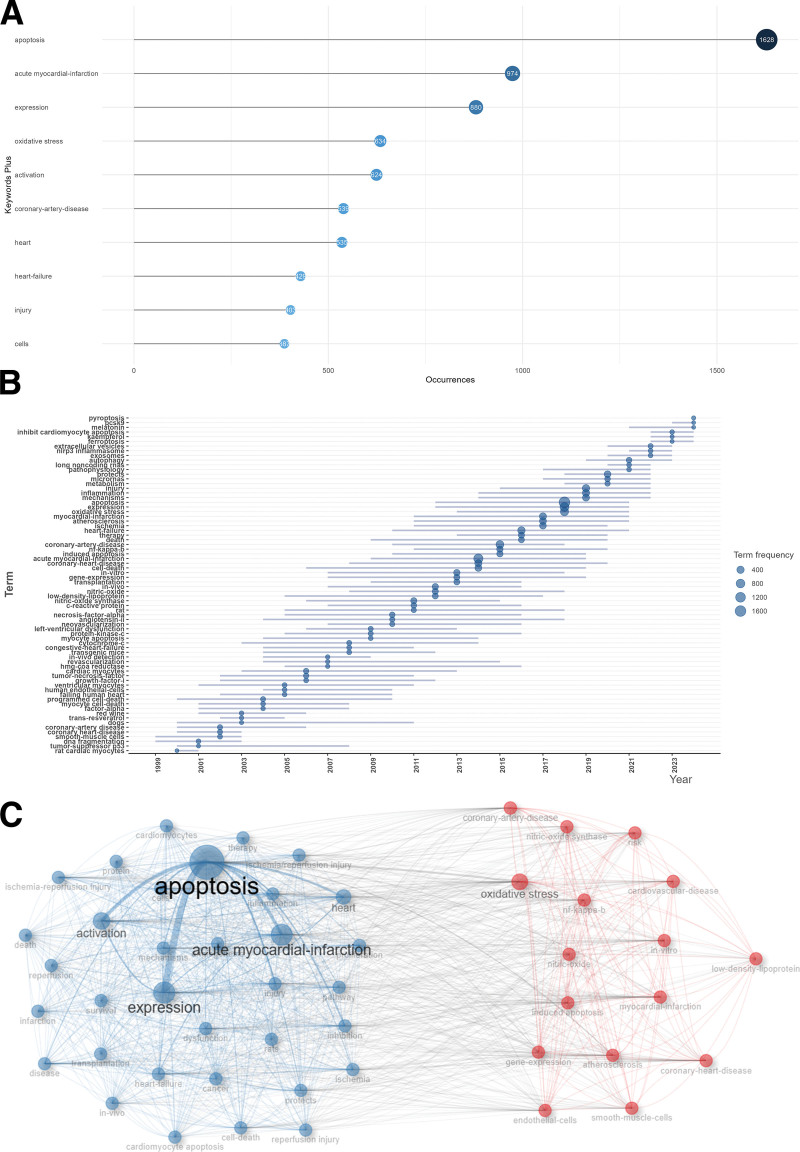
Analysis of keywords. (A) The top 10 most frequent keywords. (B) The trend topics (keyword minimum frequency > 100) over time. (C) The keywords co-occurrence network map.

The most frequent keywords included “apoptosis” with 1628 times, followed by “acute myocardial-infarction” with 974 times and “expression” with 880 times. The fourth keyword was “oxidative stress” with 634 times and the fifth was “activation” with 624 times (Fig. 6A).

As revealed by Figure 6B, terms with a frequency of more than 10 were extracted for the further analysis. Importantly, “metabolism,” “microRNAs,” “protects,” “pathophysiology,” “long noncoding RNAs,” “extracellular vesicles,” “autophagy,” “exosomes,” “nlrp3 inflammasome,” “ferroptosis,” “kaempferol,” “inhibit cardiomyocyte apoptosis,” “melatonin,” “pcsk9,” and “pyroptosis” emerged after 2020, and have continuously appeared in related articles in the last 5 years. Apoptosis following CHD may play a significant role in secondary injury mechanisms that contribute to cardiac functional impairments. Nevertheless, the molecular and cellular mechanisms underlying this process require thorough investigation. The inhibition of apoptosis is crucial for alleviating complications associated with CHD. A more profound understanding of the pathways involved in apoptosis could aid in the development of effective therapeutic interventions. The complexity of apoptosis-related pathways and potential interactions in the context of CHD highlights the need for the identification of multi-target agents and the adoption of multifaceted therapeutic strategies that prioritize safety and tolerability. It is important to note that the complexity of CHD should not be reduced to a single pathophysiological mechanism or the activation or inhibition of a specific form of cell death. Consequently, combination therapies that address various modalities of programmed cell death may offer more effective approaches for the management of CHD. There is an urgent necessity for enhanced efforts to translate current advancements into therapeutic strategies aimed at modulating cell death pathways within the realm of apoptosis and CHD.

In the analysis of relevant literature, keywords that frequently appeared together were grouped into clusters. A keyword co-occurrence network was constructed based on the top 50 most prevalent keywords, which were categorized into 2 primary clusters (Fig. 6C). The red cluster included several frequent keywords, including “oxidative stress,” “coronary-artery-disease,” “coronary-heart-disease,” “myocardial-infarction,” “atherosclerosis,” and “smooth-muscle-cells.” The blue cluster included keywords like “apoptosis,” “acute myocardial-infarction,” “expression,” “activation,” “heart,” “heart-failure,” and “injury.” The most popular hot topics involved the correlations between oxidative stress and apoptosis in CHD, as well as potential cellular and molecular mechanisms and therapeutic strategies.

### 3.6. Thematic analysis

**Figure 7.**
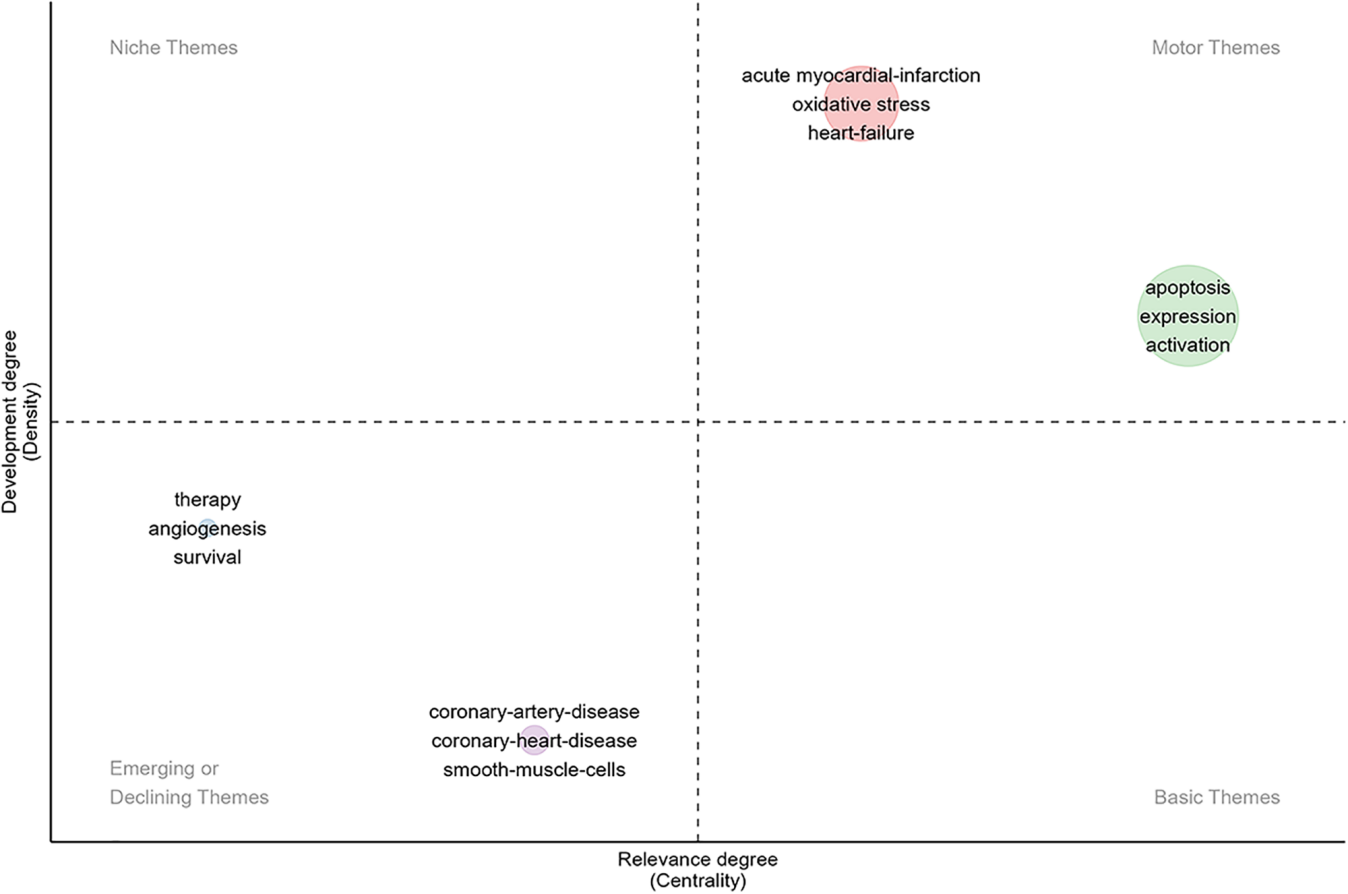
Thematic map analysis. the *X* axis is centrality while *Y* axis is density; the map is divided into 4 quadrants which are motor themes, niche themes, emerging or declining themes, and basic and transversal themes, respectively.

Four thematic clusters are displayed in Figure 7. The red cluster (AMI, oxidative stress, and heart-failure) and green cluster (apoptosis, expression, and activation) represented motor themes – important and well-developed themes. The blue cluster (therapy, angiogenesis, and survival) and purple cluster (coronary-artery-disease, coronary-heart-disease, and, smooth-muscle-cells) represented the emerging or declining themes in this field, which were important but were not adequately developed.

## 4. Discussion

### 4.1. General information

Apoptosis is a significant factor in the mechanisms underlying CHD, contributing substantially to morbidity and mortality rates in the human population. A bibliometric analysis was deemed necessary to thoroughly assess the existing research in this area, thereby providing an overview and identifying potential avenues for future investigation. In this study, we evaluated 5650 publications from the years 1994 to 2025 that pertain to apoptosis and CHD.

Annual publication number is an indicator of researchers’ interest in a particular field. Notably, research on apoptosis and CHD experienced a marked increase beginning in 2014. Specifically, between 2014 and 2015, there was a significant surge in publications, with an increase of over 100 articles, primarily focusing on the effects of certain molecules on heart failure in in vivo animal studies. This focus persisted for several years, until the exploration of the relationship between signaling pathways and cardiomyocytes became a prominent area of research, emerging as a focal point in the study of apoptosis and CHD. The field reached a peak in popularity in 2022, with annual publications exceeding 450. However, there was a decline of approximately 100 publications from 2022 to 2023.

Among the countries evaluated, China emerged as the leader in terms of the total publication number, while the United States was identified as the most influential country in this field. China was the home to various related affiliations, notably the Chinese Academy of Medical Sciences-Peking Union Medical College, followed by Harbin Medical University, Peking Union Medical College, and Chang Gung Memorial Hospital. The United States ranked second in total publications, demonstrating a relatively high incidence of SCPs and multiple-countries publications. Numerous countries collaborated to address existing challenges, with the United States exhibiting the strongest collaborative ties with both the United Kingdom and China. Notably, China displayed the highest MCP ratio, which indicated that a substantial proportion of its published articles resulted from international collaboration. Global cooperation should be promoted further in the future. In terms of related authors, Wang Y and Zhang Y were identified as the most productive authors.

**Figure 8.**
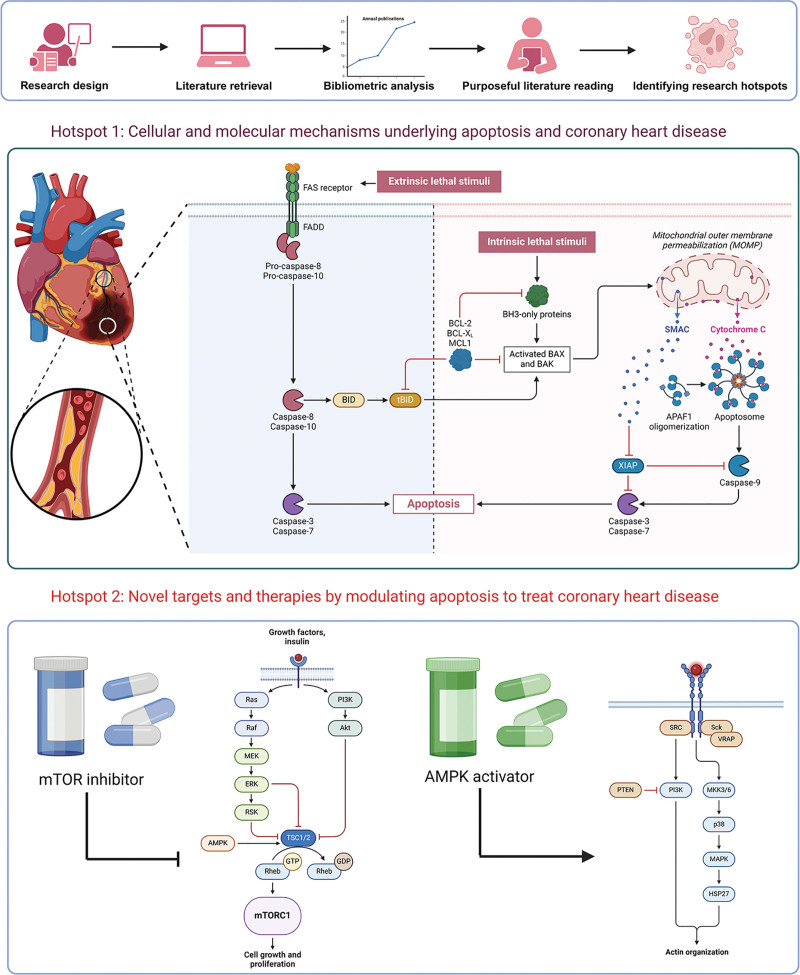
Dynamic research hotspots diagram. We have summarized the following 2 hotspots: cellular and molecular mechanisms underlying apoptosis and coronary heart disease; novel targets and therapies by modulating apoptosis to treat coronary heart disease, including the applications of mTOR inhibitor and AMPK activator.

When it comes to the journals, *Molecular Medicine Reports, International Journal of Molecular Sciences*, and *Plos One* were the journals with the highest number of publications. The most locally cited journals included *Circulation* and *Circulation Research*. We systematically summarized the current knowledge regarding the molecular mechanisms and interactions between apoptosis and CHD. We further elucidated the distinct roles of apoptosis and therapeutic strategies in myocardial ischemia and ischemia/reperfusion (I/R) injury. This study will contribute to the development of a treatment paradigm that shifts from single-target therapies to a more balanced approach addressing both apoptosis and other cell-death types in the management of CHD. The dynamic hotspots in this field were summarized as follows: cellular and molecular mechanisms underlying apoptosis and CHD; critical targets and therapies by modulating apoptosis to treat CHD. The mechanisms associated with these hotspots are illustrated by Figure 8.

### 4.2. Evidence of apoptosis after CHD

Apoptosis has been observed in animal CHD models (mostly in mice models) and human CHD, including myocardial infarction, ventricular hypertrophy, ventricular dilatation, and autoimmunity diseases.^[26]^ In heart failure, the apoptosis rate in myocytes is 232-fold increasing than the normal level, and the level of expression of B-cell lymphoma (BCL) in heart failure patients is almost doubling that of healthy samples, which is necessary for preventing cells from apoptosis.^[27]^ In human myocardial infarction, the apoptosis rate of cardiomyocytes is about 2% to 12%.^[28]^ Another study showed that a higher incidence of cardiomyocytes apoptosis was found in failed hearts of patients with ischemic cardiomyopathy compared to those with idiopathic dilated cardiomyopathy.^[29]^

### 4.3. The morphology of apoptosis in CHD

Upon the activation of apoptotic pathways, the cell undergoes a series of morphological alterations, including a reduction in size, increased cytoplasmic density, and a more compact arrangement of organelles, accompanied by chromatin condensation. Following these changes, the plasma membrane exhibits blebbing, which engulfs the densely aggregated organelles, with or without the presence of nuclear fragments, resulting in the development of apoptotic bodies. Concurrently, cytoskeletal and nuclear proteins are subjected to degradation and cleavage by caspases.^[30]^ Ultimately, apoptotic bodies are engulfed by macrophages, a process that is enhanced by the externalization of phosphatidylserine on the plasma membrane.^[31]^

The apoptotic process described above encompasses various proteins involved in cell death and phagocytosis, with caspases playing a pivotal role. Notably, caspase-3 serves as a critical convergence point for apoptosis-related signaling pathways. Upon activation, caspases typically initiate the activation of other procaspases, thereby promoting a proteolytic cascade that irreversibly amplifies the apoptotic signaling pathway.^[31]^

### 4.4. Apoptosis induced by the extrinsic pathway

Apoptosis via the extrinsic pathway is initiated by transmembrane death receptors, which belong to the tumor necrosis factor (TNF) receptor family. Key ligands and their receptors that facilitate this apoptotic process include apoptosis-stimulating fragment ligand (FasL) in conjunction with Fas receptor (FasR), TNF-α with TNF receptor 1 (TNFR1), and TNF-related apoptosis-inducing ligand (TRAIL) interacting with death receptors DR4 and DR5. These interactions enable the transmission of death signals. For instance, upon the binding of TNF-α to TNFR1 or FasL to FasR, the fas-associated death domain is activated, which can form a ligand-receptor complex.^[32]^ fas-associated death domain can activate procaspase-8, leading to the formation of a death-inducing signaling complex.^[33]^ Ultimately, caspase-8, once activated by death-inducing signaling complex, initiates the activation of caspases-3 and -7, thereby triggering the apoptotic cascade and culminating in the execution phase of apoptosis.^[34]^

### 4.5. Apoptosis induced by the intrinsic pathway

The intrinsic pathway, which is activated by mitochondrial processes, has the capacity to induce apoptosis. This pathway is frequently activated by various stimuli, such as hyperthermia and hypoxia. Such stimuli facilitate the opening of the mitochondrial permeability transition pore, and consequently enhance the release of pro-apoptotic proteins, such as Cytochrome c (Cyt c).^[35]^ Cyt c binds to and activates Apaf-1 and procaspase-9, inducing the activation of caspase-9, which subsequently promotes the activation of caspases-3 and -7.^[36,37]^

Importantly, the aforementioned mitochondrial-initiated processes are mediated by B-cell lymphoma 2 (Bcl-2) proteins.^[38]^ These proteins modulate the permeability of the mitochondrial membrane and thereby influence the release of Cyt c. Bcl-2 family proteins could be functionally categorized into anti-apoptotic and pro-apoptotic proteins. The pro-apoptotic group includes Bad, Bax, Bak, Bid, Bim, Puma, and BNIP3,^[39]^ while the anti-apoptotic group comprises Bcl-2, Bcl-x, Bcl-XL, and BAG. Among these proteins, the expression of Bax could be enhanced by the tumor suppressor protein p53, either in the mitochondria or nucleus (referred to as mp53 or np53).^[37]^ In contrast, Bcl-2 and Bcl-xL may inhibit apoptosis mediated by np53 or mp53. When the p53 expression is suppressed, both caspase-3 and Bax are inhibited in the cardiac tissue of individuals with CHD.^[40]^ Notably, there are also interactions between the intrinsic and extrinsic apoptotic pathways.^[41]^

### 4.6. Apoptosis induced by the endoplasmic reticulum (ER) stress pathway

ER stress is a significant pathway in the regulation of apoptosis.^[42,43]^ While ER stress is essential for cellular survival, it can also trigger apoptotic processes under conditions of chronic or prolonged stress.^[44]^ Cellular responses to ER stress are regulated by the unfolded protein response, which could be activated by the integral membrane proteins located in the ER.^[45]^ Prolonged activation of IRE1 may interact with the adaptor protein TNF receptor-associated factor-2 (TRAF2), subsequently initiating the c-Jun N-terminal protein kinase (JNK) pathway.^[46,47]^ Consequently, it is posited that JNK is related to IRE1-regulated apoptosis, potentially through JNK-Bad signaling pathways that are involved in ER stress.^[48]^ Notably, prior research indicated that mammalian IRE1α can bind to pro-apoptotic proteins Bak and Bax, thereby facilitating its activation. Furthermore, procaspase-12 has been shown to interact with the IRE1-TRAF2 complex, leading to the activation of caspase-12.^[49]^ Additionally, prolonged IRE1 activity may promote apoptosis through the regulated Ire1-dependent decay pathway,^[50]^ whereas the precise mechanisms governing this pathway remained unclear. Further, IRE1α can splice and activate X-box binding protein-1 (XBP1) mRNA,^[51]^ which plays a role in promoting cellular survival. Thus, IRE1α is recognized as a pivotal protein that may influence the balance between survival and apoptosis.

The protein PERK facilitates the phosphorylation of the translation initiation factor eIF2α, leading to the translation of ATF4 in pro-apoptotic processes.^[45]^ In conditions of severe ER stress, ATF4 promotes the expression level of C/EBP-homologous protein (CHOP).^[43]^ Accumulating evidence indicates that CHOP can inhibit Bcl-2.^[52]^ while promoting the expression of pro-apoptotic proteins such as Bax.^[53]^ Additionally, in oxidative stress, CHOP may activate ER oxidase 1α (ERO1α)^[54]^ and triggers calcium-dependent apoptosis via CHOP-ERO1α-IP3R1-calcium-CaMKII pathway,^[55]^ which subsequently results in the upregulation of JNK and the activation of ROS and Nox2.^[56]^ ROS activates PKR, which can enhance the expression of CHOP through a positive feedback mechanism.^[56]^ Furthermore, apoptosis induced by CaMKIIγ is associated with JNK-Fas and JNK-Cyt c signaling pathways. Additionally, during ER stress, caspase-12 and Bid have been implicated in the CHOP-ERO1α-IP3R-calcium-calpain pathway.^[57,58]^ Notably, apoptosis mediated by IRE1α and CHOP can be mitigated by a phenomenon known as pre-conditioning, wherein low levels of ER stress partially suppress the unfolded protein response prior to the activation of a robust UPR stimulus.^[59]^ These results suggested a positive relationship between ER stress-regulated and mitochondria-regulated apoptosis, which can be facilitated by Bcl-2. Moreover, ATF6 is capable of activating XBP1 mRNA, thereby exerting a protective effect.^[51]^ Correspondingly, research indicated the inhibition of ATF6 can facilitate apoptosis.^[60]^ Nevertheless, the precise mechanisms underlying ATF6-mediated apoptosis remained unclear.

### 4.7. MicroRNAs (miRNAs)-related apoptosis in CHD

MicroRNAs have been demonstrated to play an important role in the regulation of apoptosis in CHD.^[61]^ Specific miRNAs have been implicated in the pathogenesis of CHD by modulating critical genes and pathways associated with anti-apoptotic mechanisms.^[62]^ Among these, miR-320 has emerged as a particularly important miRNA in CHD, as it regulates cardiac ischemia/reperfusion injury through regulating heat-shock protein 20 (HSP20). Subsequent investigations have confirmed that miR-320 is important in mediating ischemic cardio-protection and myocardial reperfusion injury through its interaction with HSP20.^[63]^ Furthermore, traditional Chinese medicine interventions have been shown to influence apoptosis in an anti-atherosclerotic manner by modifying miRNA expression, thereby promoting cell growth, cardio-protection, and the inhibition of apoptosis. The qualitative and quantitative characteristics of certain serum-derived exosomal miRNAs in CHD patients with hyperglycemia may serve as alternative biomarkers for predicting the severity of coronary stenosis.^[63]^ Notably, 3 miRNAs – has-let-7b-5p, hsa-miR-4313, and hsa-miR-940 – have been identified at baseline as directly influencing the severity of stenosis in acute conditions affecting the left anterior descending artery (LAD).^[64]^ Moreover, miR-155 has been identified to mitigate excessive vascular remodeling in cardiovascular diseases such as CHD by modulating hematopoietic lineage-specific differentiation through immune and inflammatory pathways.^[64]^ These findings indicated that miR-155 may represent a novel therapeutic target, not only by regulating apoptosis but also by regulating various pathogenic responses linked to vascular dysfunction in the progression of CHD. Moreover, miR-30c has been identified as a microRNA that may regulate cellular proliferation and apoptosis through the Sonic hedgehog signaling pathway in cardiac myocytes.^[65]^

### 4.8. Vascular smooth muscle cells (VSMCs) apoptosis in CHD

Apoptosis of vascular smooth muscle cells (VSMCs) is a significant factor in the progression of CHD. Inflammatory cytokines, including tumor necrosis factor-alpha (TNF-α), can promote VSMC apoptosis by inducing programmed cell death through the activation of caspases, a family of enzymes that facilitate cell suicide.^[66]^ Additionally, chronic oxidative stress represents the second most detrimental factor contributing to VSMC apoptosis in CHD.^[67]^ One of the critical pathways mediating VSMC apoptosis is the intrinsic apoptotic pathway, which is regulated by mitochondrial dysfunction. This pathway is characterized by the release of cytochrome c from the mitochondria into the cytoplasm, where it binds to apoptotic protease activating factor-1 (Apaf-1) and procaspase-9, forming a complex that can activate caspase-9, subsequently triggering downstream cascades of other caspases.^[68]^ Furthermore, the extrinsic apoptotic pathway, which is activated by the interaction of death ligands with their corresponding receptors on the cell surface, also plays an important role in VSMC apoptosis.^[69]^

Inhibiting apoptotic pathways in VSMCs has demonstrated potential therapeutic benefits for CHD. For instance, a research performed by Fu et al^[70]^ revealed the blockade of BAG3 and the inhibition of microRNA-24 in VSMCs significantly suppresses the phenotypic changes associated with apoptosis, particularly in response to platelet-derived growth factor. The PI3K/Akt signaling pathway has been identified as an intrinsic mechanism that protects against VSMC apoptosis, with phosphatase and tensin homolog (PTEN) serving as a crucial regulatory molecule.^[70]^ The apoptosis of VSMCs is integral to the pathophysiology of CHD. A better understanding of the mechanisms underlying VSMC apoptosis may facilitate the development of therapeutic strategies aimed at mitigating cardiovascular events and enhancing patient outcomes.^[4]^

### 4.9. Apoptosis of cardiomyocytes in CHD

The apoptosis of cardiomyocytes is triggered by various stressors, such as ischemia, reperfusion injury, and oxidative stress, all of which play a significant role in the pathogenesis of CHD. This process results in myocyte death, initiating a cascade of cellular events that contribute to ischemia due to reduced oxygen and blood flow to the heart. During ischemic conditions, the deficiency of oxygen and nutrients induces metabolic alterations, leading to the accumulation of toxic metabolites and a decrease in ATP production, which subsequently activates apoptotic pathways.^[19,27]^ Reperfusion, necessary for restoring blood flow following ischemia, paradoxically results in the generation of reactive oxygen species (ROS), further promoting apoptosis by serving as a critical modulator that compromises mitochondrial integrity.^[20]^

Both extrinsic and intrinsic apoptotic pathways collaboratively contribute to myocyte apoptosis in CHD through various molecular mechanisms. The intrinsic pathway is predominantly dependent on mitochondrial function and culminates in the release of cytochrome c, which activates caspases.^[71]^ Conversely, the extrinsic pathway involves death receptors that, upon binding with specific ligands, initiate intracellular signaling cascades through caspase activation.^[72]^ Research has shown that cardiac myocytes that inhibit apoptosis exhibit reduced infarct size and beneficial effects on cardiac function. Transgenic mice that overexpress the anti-apoptotic protein Bcl-2 demonstrate decreased myocyte apoptosis and improved survival following ischemic injury.^[73]^ Therefore, interventions aimed at targeting apoptotic pathways may represent a promising therapeutic strategy to mitigate myocyte loss and preserve cardiac function in patients with CHD.

### 4.10. Excessive apoptosis can promote myocardial ischemia injury

Excessive apoptosis has been identified in blood samples as well as cardiac tissues of individuals experiencing myocardial ischemia.^[74]^ Prior research indicated apoptosis represents the initial mechanism of cardiomyocytes death, with a tendency to occur at the peripheries of the infarcted area.^[75]^ The presence of cleaved caspase-3 p17 peptide, which serves as terminal effector caspase in apoptotic processes, is elevated within individuals with ST-segment elevation myocardial infarction (STEMI).^[76]^ Furthermore, p53, acting as an upstream regulator of Bax, accumulates in cardiac tissue following myocardial infarction and is found to be elevated in the monocytes of CHD patients, alongside heightened oxidative stress and apoptotic responses.^[77]^ Moreover, TNF-α and its receptor TNFR1 are markedly elevated and may serve as predictors of infarct size in STEMI cases.^[78]^ Notably, TNFR1 exhibits a strong correlation with cardiovascular outcomes.^[79]^ Though the soluble Fas (sFas) receptor is significantly increased in individuals with AMI, the levels of sFas and sFas ligand (sFasL) in peripheral blood do not show a consistent association with infarct size or left ventricular (LV) dysfunction in individuals with STEMI postpercutaneous coronary intervention.^[80]^ Contrarily, some studies have reported decreased levels of TRAIL in AMI patients^[81]^ or CHD patients.^[82]^ Further1q, TRAIL levels have been found to be inversely related to the severity of CHD,^[82]^ acute coronary syndrome,^[83]^ and cardiovascular events after AMI.^[81]^

### 4.11. Enhanced apoptosis may promote I/R injury

The I/R process can result in the generation of excessive ROS, resulting in cellular damage and cardiovascular function impairments, which may contribute to the apoptosis of cardiomyocytes. Research indicated that the upregulated expression of cardiac-specific caspase-3 exacerbates infarct size, which may heighten the risk of mortality following I/R injury.^[84]^ Notably, the Fas signaling pathway serves as a pivotal regulator of I/R-mediated apoptosis of cardiomyocytes.^[85]^ Additionally, levels of TNF-α and TRAIL were identified to be increased at the initiation of reperfusion in ischemia/reperfusion models.^[86]^ In terms of intrinsic apoptosis, the upregulation of Bax has been observed in ischemic myocardial tissues, and inhibited apoptosis has been shown to facilitate the amelioration of I/R injury.^[87]^ Furthermore, cardiac-specific upregulation of Bcl-2 can mitigate apoptosis of cardiomyocytes and reduce infarct following I/R injury.^[73]^

### 4.12. Promoted apoptosis can accelerate postinfarction cardiac remodeling

Cardiac remodeling is related to elevated LV volumes and impaired function. Further, a clinical investigation has demonstrated elevated levels of myocardial apoptosis contribute to LV remodeling.^[88]^ Furthermore, apoptosis is associated with progressive signals such as thickening of the posterior wall and myocardial fibrosis.^[89]^ The possible mechanisms involved do not appear to be associated with the extrinsic apoptotic pathway, as there are minimal associations between soluble TNF receptors (sTNFR1, sTNFR2), soluble Fas (sFas), and soluble Fas ligand (sFasL) with remodeling metrics in individuals diagnosed with ST-elevation myocardial infarction (STEMI) after percutaneous coronary intervention.^[74]^ Additional experimental research indicated that apoptosis of cardiomyocytes induced by myocardial infarction is mediated through the CHIP-p53 pathway; repression of p53 has been shown to mitigate myocardial apoptosis and ventricular remodeling.^[90]^ The aforementioned studies suggested that apoptosis played a role in accelerating cardiac remodeling following infarction.

### 4.13. Enhanced apoptosis may contribute to coronary atherosclerosis

Atherosclerosis serves as fundamental pathophysiological mechanism underlying coronary vasculature in CHD. A hallmark of atherosclerosis is the aberrant apoptosis of VECs, macrophages, and VSMCs, which can induce the development and instability of atherosclerotic plaques.^[91,92]^ Evidence suggested enhanced apoptosis can exert both harmful and protective influences on coronary atherosclerosis, contingent upon the specific types of vascular cells involved.^[91,93,94]^ Notably, oxidant hypochlorous acid has been implicated in inducing DNA damages and inflammatory responses, which are associated with ER stress-mediated apoptosis. Further, 8-chloro-adenosine has been demonstrated to induce prolonged ER stress, thereby enhancing apoptosis of human coronary artery endothelial cells (HCAECs).^[93]^ The apoptotic cell death of HCAECs subsequently exacerbates endothelial injury, potentially initiating the progression of coronary atherosclerosis. Furthermore, apoptosis of macrophage during ER stress is regulated by the CHOP-Bax pathway, which can lead to the rupture of atherosclerotic plaques, thereby increasing the risk of acute coronary syndrome. Further, research has indicated that inflammation-induced apoptosis is modulated by interleukin-10 (IL-10), which can inhibit the apoptosis of VSMCs via the JAK2-STAT3 signaling pathway during atherosclerosis development.^[94]^ Intrinsic apoptosis pathway may also play a role, as JAK2-STAT3 activation can enhance Bcl-2 expression and inhibit programmed cell death.^[95]^

Clinical studies have further elucidated the relationship between sTRAIL-R2, sTRAIL, and apoptosis of plaque cells, as well as plaque inflammation. Elevated levels of sTRAIL and sTRAIL-R2 have been identified in symptomatic carotid plaques, and patients exhibiting higher plasma concentrations of sTRAIL-R2 are at a higher risk for future cardiovascular events.^[96]^ Additionally, an investigation involving 520 participants revealed the Arg/Arg genotype of the p53 gene is least prevalent among those with left main coronary artery diseases, suggesting a potential negative correlation between this genetic polymorphism and the progression of atherosclerosis, as well as cardiovascular prognosis.^[97]^ Collectively, aberrant apoptosis is not only fundamental to the development and advancement of coronary atherosclerosis, but it may also function as an early indicator of unfavorable cardiovascular outcomes by evaluating biomarkers associated with apoptosis.

### 4.14. Therapeutic strategies of apoptosis for CHD

Excessive apoptosis is a contributing factor in the pathogenesis of CHD, leading to the consideration of apoptosis suppression as a promising therapeutic strategy for this condition. Several pharmacological agents used in the treatment of CHD have been recognized for the ability to inhibit apoptosis. For instance, olmesartan can suppress Fas-mediated apoptosis and enhance LV remodeling postmyocardial infarction.^[98]^ Similarly, Simvastatin can decrease apoptosis of cardiomyocytes and enhance cardiac function after myocardial infarction. This effect is related to a suppression of caspase-3 and Bax, as well as an upregulation of Bcl-2.^[99]^ Notably, β-adrenergic receptor blockers and angiotensin-converting enzyme inhibitors have also been identified to inhibit myocardial apoptosis.^[100]^

Moreover, recent research has focused on pharmacological compounds that may serve as specific apoptosis inhibitors. For example, salubrinal has been shown to selectively inhibit the dephosphorylation of eIF2α, promoting cell survival by mitigating prolonged ER stress.^[101]^ Additionally, EN460 and QM295, 2 selective ERO1 inhibitors, have been reported to protect mouse embryonic fibroblasts from ER stress.^[102]^ Furthermore, the chemical chaperone 4-phenyl butyric acid has been utilized to alleviate pathological ER stress, though the therapeutic mechanisms related to apoptosis remained unclear.^[103]^ Additionally, 17-allylamino-17-demethoxy geldanamycin (17-AAG) has been demonstrated to inhibit p53 and reduce the apoptosis of myocardial cells. While NF-κB has been identified as a significant activator of apoptosis through its interaction with p53 and is negatively regulated by Bcl-2, it has indicated that the administration of M2b macrophages significantly mitigates early myocardial ischemia/reperfusion injury by inhibiting NF-κB signaling.^[104]^

Nevertheless, despite TNF-α being a crucial activator of extrinsic apoptosis, the use of its antagonist, etanercept, has not yielded the anticipated results in patients with AMI.^[105]^ Additionally, patients with psoriasis have shown benefits from anti-TNF-α treatments.^[106]^ Not all apoptosis-related regulators are suitable candidates for therapeutic intervention, and those identified in cellular or animal studies require further validation through clinical trials. Given that several apoptosis-related regulators are implicated in multiple pathways, targeting pro-apoptotic Bcl-2 family members, along with the applications of drug combination, may represent a more effective therapeutic approach.^[50]^

### 4.15. Future directions

Despite the significant progress made in understanding the role of apoptosis in CHD, several areas remain underexplored and warrant further investigation. One such area is the role of novel biomarkers in apoptosis and CHD. Recent studies have identified miRNAs and lncRNAs as potential biomarkers for myocardial ischemia-reperfusion injury,^[107,108]^ but their specific roles and mechanisms in CHD are not yet fully understood. Future research should focus on elucidating the functions of these biomarkers and exploring their potential as therapeutic targets. Additionally, the development of multi-target therapeutic strategies is crucial for addressing the complexity of CHD. Current treatments often target single pathways, but the interplay between apoptosis and other cellular processes, such as autophagy and inflammation, suggests that combination therapies may be more effective.^[61]^ Further research is needed to identify safe and effective multi-target agents that can modulate apoptosis and other cell-death pathways in CHD.

Another emerging area of interest is the exploration of the interactions between apoptosis and other cellular processes in CHD. For example, the crosstalk between apoptosis and autophagy has been shown to play a significant role in myocardial ischemia-reperfusion injury, but the underlying mechanisms are still not fully understood.^[61]^ Future studies should focus on unraveling these interactions and identifying potential therapeutic targets that can modulate both processes. Additionally, the role of apoptosis in the progression of coronary atherosclerosis, particularly in the context of vascular smooth muscle cells, requires further investigation. Understanding the specific mechanisms by which apoptosis contributes to plaque instability and rupture could lead to the development of novel therapeutic strategies for preventing acute coronary syndromes.

In summary, while significant progress has been made in understanding the role of apoptosis in CHD, several areas remain underexplored. Future research should focus on elucidating the roles of novel biomarkers, developing multi-target therapeutic strategies, and exploring the interactions between apoptosis and other cellular processes.

### 4.16. Limitations

Several limitations existed in our work. First, our study was limited to the WOSCC database. Therefore, we could not guarantee that all related documents were involved in this study. Second, the database is constantly updated so that several latest articles were not included in this study.

## 5. Conclusion

This bibliometric analysis provided a comprehensive overview of the current research trends in the field of apoptosis and CHD. Our study identified key areas of research, including the cellular and molecular mechanisms underlying apoptosis in CHD. Our results highlighted the importance of apoptosis as a therapeutic target for CHD and suggested potential avenues for future research. Future studies should focus on elucidating the roles of novel biomarkers, developing multi-target therapeutic strategies, and exploring the interactions between apoptosis and other cellular processes.

## Acknowledgments

We thank the Web of Science™ (WOS) team for using their data.

## Author contributions

**Conceptualization:** Wanjun Jin, Qi Yu, Siqiao Wang.

**Data curation:** Wanjun Jin, Qi Yu, Siqiao Wang.

**Formal analysis:** Wanjun Jin, Qi Yu, Siqiao Wang.

**Funding acquisition:** Wanjun Jin, Qi Yu, Siqiao Wang.

**Investigation:** Wanjun Jin, Qi Yu, Siqiao Wang.

**Methodology:** Wanjun Jin, Qi Yu, Siqiao Wang.

**Project administration:** Wanjun Jin, Qi Yu, Siqiao Wang.

**Resources:** Wanjun Jin, Qi Yu, Siqiao Wang.

**Software:** Wanjun Jin, Qi Yu, Siqiao Wang.

**Supervision:** Wanjun Jin, Qi Yu, Siqiao Wang.

**Validation:** Wanjun Jin, Qi Yu, Siqiao Wang.

**Visualization:** Wanjun Jin, Qi Yu, Siqiao Wang.

**Writing – original draft:** Wanjun Jin, Qi Yu, Siqiao Wang.

**Writing – review & editing:** Wanjun Jin, Qi Yu, Siqiao Wang.
